# Clinical versus radiographical factors associated with hepatocellular carcinoma diagnosis in high-risk patients: sizes matter

**DOI:** 10.2144/fsoa-2021-0108

**Published:** 2022-02-02

**Authors:** Tanita Suttichaimongkol, Manoon Mitpracha, Kawin Tangvoraphonkchai, Phuangphaka Sadee, Kittisak Sawanyawisuth, Wattana Sukeepaisarnjaroen

**Affiliations:** 1 Department of Medicine, Faculty of Medicine, Khon Kaen University, Khon Kaen, 40002, Thailand; 2 Department of Medicine, Division of Gastroenterology, Khon Kaen Regional Hospital, Khon Kaen, 40002, Thailand

**Keywords:** cirrhosis, hepatitis virus, size

## Abstract

**Aim:**

This study aimed to evaluate if clinical or radiographic findings can be used for hepatocellular carcinoma (HCC) diagnosis particularly in high-risk patients.

**Methods:**

This was a prospective study and evaluated factors associated with HCC.

**Results:**

There were 260 patients met the study criteria: 219 patients (84.23%) were HCC. Two factors significantly associated with HCC: portal vein invasion and the largest mass size. The cutoff point for the largest mass size of 2 cm or over gave sensitivity and specificity for HCC of 83.56 and 87.80%, respectively.

**Conclusion:**

Portal vein invasion and the largest liver mass of 2 cm or over may be diagnostic factors for HCC in high-risk patients, while clinical factors were not suggestive for HCC.

## Plain language summary

Radiographic findings are suggestive for liver cancer and can be used to diagnose liver cancer in patients at risk for liver cancer.

Hepatocellular carcinoma (HCC) is a common cancer with a rising in its trend. In the USA, the incidence of HCC is three-times higher than the past three decades with at least 20,000 new cases [1]. The incidence of HCC has sex and race disparities: the highest rate in Asians, particularly Chinese men, with the incidence of 18.6/100,000 population [1–3]. A report from Germany found that HCC in males is four-times more common than HCC in females [4]. Patients with HCC tend to have end of life care in hospitals with a proportion of 15.2% of patients with the short survival time of 1.8 months in advanced stage if left untreated [5,6].

Risk factors include cirrhosis and hepatitis B/C virus varied among countries [3,7,8]. In India, cirrhosis was found in 99.2% of patients with HCC, while hepatitis B virus infection was found in 81.3% [7,8]. The study from Germany found HCC related with hepatitis virus in 51.9% of patients [4]. Serum alpha-fetoprotein (AFP) is used to diagnose HCC as well as other biomarkers such as protein induced by vitamin K absence-II or antagonist (PIVKA-II) [7,9]. Additional to pathological diagnosis, computed tomography (CT) or MRI can be used to diagnosis HCC [10,11]. The sensitivity of these radiographic modalities was between 65 and 79%. This study aimed to evaluate if clinical or radiographic findings can be used for HCC diagnosis, particularly in high-risk patients.

## Methods

This was a prospective study conducted at the University Hospital, Khon Kaen University, Thailand. The inclusion criteria were patients with an age of 18 years or over with a high risk for HCC. The high risk for HCC was defined by the American Association for the Study of Liver Disease guideline for HCC management [12]: cirrhosis or presence of liver nodule (s) of 1 cm or over in size. Those with pregnancy, obstructive jaundice, vitamin K or warfarin administration and presence of extrahepatic malignancy were excluded.

Data were collected as follows: baseline characteristics, laboratory results and radiographic findings. Baseline characteristics included age, sex, etiology of cirrhosis, comorbid diseases and the Child–Pugh score for cirrhosis. Laboratory tests in the study were platelet count, serum creatinine, prothrombin time, liver function test and AFP. Radiographic findings were numbers of liver mass, the largest mass size (cm) and portal vein invasion. Liver masses were measured by diameter by official radiologists who reported independently and blindly to clinical data. Portal vein invasion defined by filling defect in portal vein evidence by CT. The HCC diagnosed by either confirmation by pathological findings or CT findings of arterial hypervascularity followed by washout of contrast at venous and/or delayed phase [12]. An MRI was used as an additional tool for HCC diagnosis.

### Sample size calculation

The previous study found vascular invasion of 31% [5]. An expected vascular invasion in patients with high-risk for HCC of 50%. With a confidence of 95% and power of 90%, the estimated sample size was 276 patients.

### Statistical analyses

Patients were categorized by the presence of HCC: “HCC” or “non-HCC” group. Descriptive statistics were used to calculate differences between these two groups. Factors associated with HCC were analyzed by multivariate logistic regression analysis. Unadjusted/adjusted odds ratio (OR) and its 95% CI of each predictor were reported. The final model for predictive of HCC was tested for goodness of fit by the Hosmer–Lemeshow method. A numerical predictor for HCC was computed for appropriate diagnostic cutoff point by a receiver-operating characteristic (ROC) curve. Results of the numerical predictors were shown area under ROC curve with the 95% CI and sensitivity and specificity for best cutoff point. All statistical analyses were performed using STATA software version 10.1 (TX, USA).

## Results

**Figure 1.**
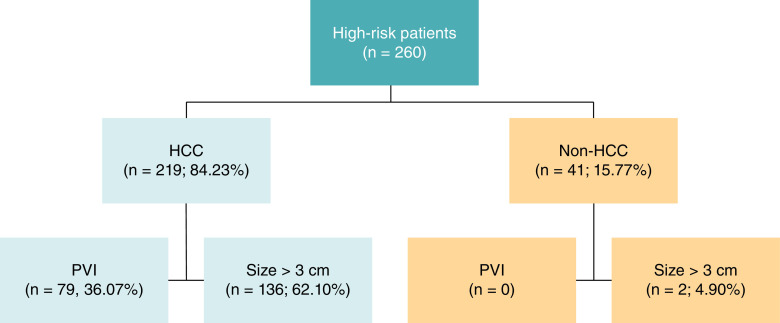
Study flow chart of patients with high risk for hepatocellular carcinoma and numbers of patients with portal vein invasion and size of over 3 cm. HCC: Hepatocellular carcinoma; PVI: Portal vein invasion.

During the study period, there were 260 patients that met the study criteria. Of those, 219 patients (84.23%) were categorized into the HCC group. The diagnosis for non-HCC group included a dysplastic nodule (25 patients), regenerative nodule (eight patients), hemangioma (five patients), liver cyst (one patient), fibronodular hyperplasia (one patient) and hepatic adenoma (one patient). For baseline characteristics, only proportion of cirrhosis classification was different between both the groups (p = 0.036). The HCC group had higher proportion of patients with Child–Pugh B and C (19.60 vs 4.90%) as shown in Table 1. Regarding laboratory results, only platelet count and serum creatinine level were not significantly different between both the groups (Table 2). The HCC group had more proportions of patients with the largest mass size over 3 cm and portal vein invasion than the non-HCC group (62.10 vs 4.90% and 36.07 vs 0%, respectively) as shown in Figure 1.

**Table 1.** Baseline characters of patients with high risk for hepatocellular carcinoma (HCC) categorized by diagnosis of HCC.

Factors	HCC (n = 219)	Non-HCC (n = 41)	p-value
Age, years	59 (54–64)	57 (52–61)	0.143
Male sex, n (%)	181 (82.60)	30 (73.20)	0.154
Etiology			0.686
HBV	83 (37.90)	15 (36.60)	
HCV	94 (42.90)	20 (48.00)	
HBV plus HCV	4 (1.80)	2 (4.90)	
NAFLD	13 (5.90)	1 (2.40)	
ALD	24 (11.00)	3 (7.30)	
AIH	1 (0.50)	0	
Co-morbid diseases			0.471
None	164 (74.90)	29 (70.70)	
Diabetes	29 (13.20)	9 (22.00)	
Hypertension	10 (4.60)	2 (4.90)	
Cirrhosis	217 (99.09)	39 (95.12)	0.036
Child-Pugh score A	174 (79.50)	37 (90.20)	
Child-Pugh score B	34 (15.50)	2 (4.90)	
Child-Pugh score C	9 (4.10)	0	

HBV: Hepatitis B virus; HCC: Hepatocellular carcinoma; HCV: Hepatitis C virus; NAFLD: Nonalcoholic fatty liver disease; ALD: Alcoholic liver disease; AIH: Autoimmune hepatitis.

**Table 2.** Laboratory results of patients with high risk for hepatocellular carcinoma (HCC) categorized by diagnosis of HCC.

Factors	HCC (n = 219)	Non-HCC (n = 41)	p-value
Platelet x 10^3^/mm^3^	149 (101–224)	130 (110–192)	0.388
Creatinine (mg/dl)	0.94 (0.79–1.16)	0.92 (0.81–1.10)	0.695
Prothrombin time (s)	12.50 (11.60–13.50)	11.70 (11.00–12.50)	<0.001
Albumin (g/dl)	3.80 (3.30–4.30)	4.40 (3.70–4.70)	<0.001
Bilirubin (mg/dl)	1 (0.70–1.80)	0.70 (0.60–1.00)	0.003
Alanine aminotransferase (U/l)	55 (31–88)	32 (21–72)	0.005
Aspartate transaminase (U/l)	82 (45–145)	42 (23–67)	<0.001
Alpha-fetoprotein (IU/ml)	118.60 (10.20–3326.60)	4.1 (1.90–14.60)	<0.001
Radiographic findings			
Number of liver nodules, n (%)			0.012
1	129 (58.90)	27 (65.90)	
2	44 (20.10)	2 (4.90)	
3	7 (3.20)	5 (12.20)	
≥4	39 (17.80)	7 (17.10)	
Largest size, n (%)			<0.001
≤3 cm	83 (37.90)	39 (95.10)	
>3 cm	136 (62.10)	2 (4.90)	
Portal vein invasion, n (%)			<0.001
No invasion	140 (63.93)	41 (100)	
Main portal vein invasion	56 (25.57)	0	
Nonmain portal vein invasion	23 (10.50)	0	

Data presented as median (range) unless indicated otherwise.

HCC: Hepatocellular carcinoma.

**Figure 2.**
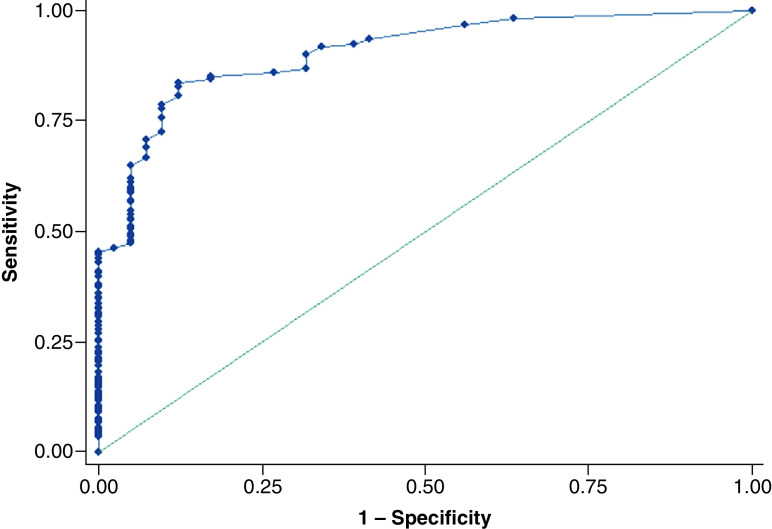
A receiver-operating characteristic curve of largest mass size for hepatocellular carcinoma in patients with high risk for hepatocellular carcinoma.

The predictive model for HCC showed that two factors were significantly associated with HCC: portal vein invasion and the largest mass size. Portal vein invasion perfectly predicted HCC: OR and 95% CI were not calculated. The adjusted OR (95% CI) for the largest mass size over 3 cm was 3.37 (1.89, 6.02) as shown in Table 3. The final predictive model has Hosmer–Lemeshow Chi square of 13.73 (p = 0.06). The cutoff point for the largest mass size of 2 cm or over gave sensitivity and specificity for HCC of 83.56 and 87.80% with the area under the ROC curve of 90.48% (95% CI: 85.97–94.98) as shown in Figure 2.

**Table 3.** Factors predictive of hepatocellular carcinoma in patients with high risk for hepatocellular carcinoma.

Factors	Unadjusted odds ratio (95% CI)	Adjusted odds ratio (95% CI)
Age	1.03 (0.99, 1.08)	1.05 (0.99, 1.01)
Sex	1.74 (0.80, 3.78)	1.65 (0.51, 5.31)
HBV or HCV infection	0.51 (0.17, 1.53)	0.72 (0.17, 3.04)
Child–Pugh score	1.25 (0.65, 2.37)	2.22 (0.30, 16.49)
Prothrombin time	1.62 (1.20, 2.18)	1.38 (0.86, 2.23)
Albumin	0.32 (0.18, 0.58)	0.92 (0.35, 2.40)
Bilirubin	1.97 (1.14, 3.39)	1.23 (0.76, 1.99)
ALT	1.01 (1.00, 1.02)	1.00 (0.98, 1.01)
AST	1.01 (1.00, 1.02)	0.99 (0.99, 1.01)
AFP	1.00 (1.00, 1.00)	1.00 (0.99, 1.01)
Number of mass	0.99 (0.74, 1.33)	0.78 (0.51, 1.18)
Largest mass size, cm	3.84 (2.23, 6.59)	3.37 (1.89, 6.02)

HBV: Hepatitis B virus; HCV: Hepatitis C virus; ALT: Alanine aminotransferase; AST: Aspartate transaminase; AFP: Alpha-fetoprotein.

## Discussion

This prospective study showed that radiographic findings were more predictive for HCC than other clinical findings: portal vein invasion and the largest mass size.

As previously reported, cirrhosis was the most common risk factor for HCC in this study (98.46%) but somewhat lower than the study from India (99.2%) [7]. Hepatitis B and C viruses were also common in this study (83.84%). Etiologies of HCC were comparable with the previous studies from Asian countries: India and Vietnam but different from the European country Germany [4,7,8].

Even though there were several factors associated with HCC both clinical and radiographic factors (Tables 1 & 2), only portal vein invasion and the largest mass size were independently associated with HCC after being adjusted with other factors (Table 3). Portal vein invasion, a sign for HCC staging, is an indicator for stage IIIB in TNM staging system and prognostic factor [13,14]. Additionally, portal vein thrombosis was associated with survival and treatment [15]. Diagnosis of portal vein thrombosis may be better if using a diffusion-weighted MRI [16]. This study found that filling defect at portal vein by CT was an indicator for HCC diagnosis. However, this filling defect may be either portal vein thrombosis or portal vein invasion. Note that rate of portal vein invasion in this study was found in 36.10%, which was comparable with the previous study (31%) [5].

As previously reported, average HCC mass size was 5.3 cm [7]. In this study, we also found that most of patients with HCC had the largest size of over 3 cm (62.10%) as shown in Table 2. This factor was also independently indicated HCC diagnosis with good sensitivity and specificity if the largest size of mass was 2 cm or over (Figure 1). As stated by the guidelines, liver mass size over 1 cm by surveillance is high-risk for HCC [12,17]. This study adds that if the size of over 2 cm, the risk for HCC diagnosis was 3.37-times higher in high-risk patients (Table 3).

There are some limitations in this study. First, as HCC is a disparity disease. The results of this study may be applied for those with similar causes of HCC. Second, some factors were not studied such as PIVKA-II. Third, the diagnosis of HCC was made mainly by typical CT [12]. Those with atypical CT findings may be missed. However, 47 patients and five patients underwent an MRI and biopsy to define HCC diagnosis. Additionally, the diagnosis non-HCC group was shown in the results. Finally, the results may not be specific to any causes of HCC as there were several causes of cirrhosis/HCC enrolled (Table 1).

## Conclusion

In conclusion, portal vein invasion and the largest liver mass of 2 cm or over may be diagnostic factors for HCC in high-risk patients, while clinical factors were not suggestive for HCC.

**Summary points**

- Radiographic findings are more suggestive for hepatocellular carcinoma (HCC) than clinical factors.
- Portal vein invasion is suggestive for diagnosis of HCC in high-risk patients.
- Liver mass of 2 cm or over is suggestive for diagnosis of HCC in high-risk patients with sensitivity of 83.56%.

## References

[1] Ha J, Chaudhri A, Avirineni A, Pan J-J. Burden of hepatocellular carcinoma among hispanics in South Texas: a systematic review. Biomark. Res. 5, 15 (2017). 28439416 10.1186/s40364-017-0096-5 PMC5399820

[2] Pham C, Fong T-L, Zhang J, Liu L. Striking racial/ethnic disparities in liver cancer incidence rates and temporal trends in California, 1988–2012. J. Natl Cancer Inst. 110(11), 1259–1269 (2018). 29617913 10.1093/jnci/djy051 PMC7191878

[3] Ajayi F, Jan J, Singal AG, Rich NE. Racial and sex disparities in hepatocellular carcinoma in the USA. Curr. Hepatol. Rep. 19(4), 462–469 (2020). 33828937 10.1007/s11901-020-00554-6 PMC8020839

[4] Kubicka S, Rudolph KL, Hanke M Hepatocellular carcinoma in Germany: a retrospective epidemiological study from a low-endemic area. Liver 20(4), 312–318 (2000). 10959810 10.1034/j.1600-0676.2000.020004312.x

[5] Cabibbo G, Maida M, Genco C Natural history of untreatable hepatocellular carcinoma: a retrospective cohort study. World J. Hepatol. 4(9), 256–261 (2012). 23060970 10.4254/wjh.v4.i9.256 PMC3468702

[6] Truitt K, Khan SS, Gregory DL, Chuzi S, VanWagner LB. Deaths from hepatocellular carcinoma are more likely to occur in medical facilities than deaths from other cancers: 2003–2018. Liver Int. 41(7), 1489–1493 (2021). 33932082 10.1111/liv.14915 PMC8822953

[7] Sood A, Midha V, Goyal O, Goyal P, Sood N, Sharma SK. Profile of hepatocellular carcinoma in a tertiary care hospital in Punjab in northern India. Indian J. Gastroenterol. 33(1), 35–40 (2014). 24222369 10.1007/s12664-013-0373-7

[8] Le VQ, Nguyen VH, Nguyen VH Epidemiological characteristics of advanced hepatocellular carcinoma in the northern region of Vietnam. Cancer Control. 26(1), 1073274819862793 (2019). 31290350 10.1177/1073274819862793 PMC6620729

[9] Feng H, Li B, Li Z, Wei Q, Ren L. PIVKA-II serves as a potential biomarker that complements AFP for the diagnosis of hepatocellular carcinoma. BMC Cancer 21(1), 401 (2021). 33849479 10.1186/s12885-021-08138-3 PMC8045263

[10] Yu NC, Chaudhari V, Raman SS CT and MRI improve detection of hepatocellular carcinoma, compared with ultrasound alone, in patients with cirrhosis. Clin. Gastroenterol. Hepatol. 9(2), 161–167 (2011). 20920597 10.1016/j.cgh.2010.09.017

[11] Wang G, Zhu S, Li X. Comparison of values of CT and MRI imaging in the diagnosis of hepatocellular carcinoma and analysis of prognostic factors. Oncol. Lett. 17(1), 1184–1188 (2019). 30655882 10.3892/ol.2018.9690 PMC6312947

[12] Bruix J, Sherman M. American Association for the Study of Liver Diseases. Management of hepatocellular carcinoma: an update. Hepatology 53(3), 1020–1022 (2011). 21374666 10.1002/hep.24199 PMC3084991

[13] Cannella R, Taibbi A, Porrello G, Dioguardi Burgio M, Cabibbo G, Bartolotta TV. Hepatocellular carcinoma with macrovascular invasion: multimodality imaging features for the diagnosis. Diagn. Interv. Radiol. 26(6), 531–540 (2020). 32990243 10.5152/dir.2020.19569 PMC7664740

[14] Shindoh J, Andreou A, Aloia TA Microvascular invasion does not predict long-term survival in hepatocellular carcinoma up to 2 cm: reappraisal of the staging system for solitary tumors. Ann. Surg. Oncol. 20(4), 1223–1229 (2013). 23179993 10.1245/s10434-012-2739-y PMC3856190

[15] Mähringer-Kunz A, Steinle V, Kloeckner R The impact of portal vein tumor thrombosis on survival in patients with hepatocellular carcinoma treated with different therapies: a cohort study. PLoS ONE 16(5), e0249426 (2021). 33961627 10.1371/journal.pone.0249426 PMC8104403

[16] Catalano OA, Choy G, Zhu A, Hahn PF, Sahani DV. Differentiation of malignant thrombus from bland thrombus of the portal vein in patients with hepatocellular carcinoma: application of diffusion-weighted MR imaging. Radiology 254(1), 154–162 (2010). 20032150 10.1148/radiol.09090304

[17] Marrero JA, Kulik LM, Sirlin CB Diagnosis, staging, and management of hepatocellular carcinoma: 2018 Practice Guidance by the American Association for the Study of Liver Diseases. Hepatology 68(2), 723–750 (2018). 29624699 10.1002/hep.29913

